# 618. Impact of Burn Etiology on Psychiatric Outcomes Following Burn Injury

**DOI:** 10.1093/jbcr/irag033.432

**Published:** 2026-04-06

**Authors:** Aidan R Carroll, Philong Nguyen, Joshua Wang, Luka Zrnic, Ryan Liang, Sudhanvan Iyer, Kamryn K Baker, Noelle Gonzalez, Gabriela Villa, Yousef Tanas, Jong Lee

**Affiliations:** University of Texas Medical Branch, Galveston, TX; University of Texas Medical Branch, Galveston, TX; University of Texas Medical Branch, Galveston, TX; University of Texas Medical Branch, Galveston, TX; University of Texas Medical Branch, Galveston, TX; University of Texas Medical Branch, Galveston, TX; McGovern Medical School at University of Texas Health Science Center at Houston, Houston, TX; University of Texas Medical Branch, John Sealy School of Medicine, Galveston, TX; University of Texas Medical Branch, Galveston, TX; Houston Methodist Hospital, Houston, TX; Division of Burn and Trauma Surgery, Department of Surgery, University of Texas Medical Branch, Galveston, Galveston, TX

## Abstract

**Introduction:**

Burn injuries are linked to long-term psychiatric morbidity, but the influence of burn type is not well defined. While prior studies highlight burn depth and size as major predictors, few have directly compared psychiatric outcomes across etiologies. This retrospective cohort study uses a large multicenter database to evaluate psychiatric morbidity following electrical, flame, and scald burns in adult burn patients.

**Methods:**

Methods: TriNetX Research Network was queried for patients ages 18 years and older who sustained burn injuries between 2003 and 2023. Patients were divided into three cohorts based on burn injury type, as identified by ICD-10 codes: electrical, flame, or scald. Patients were matched 1:1 using propensity score matching based on demographics, body mass index, substance use disorder, comorbidities, antidepressant use, preexisting psychiatric diagnoses, and total surface area burn. The primary outcomes included anxiety, depression, posttraumatic stress disorder (PTSD), adjustment disorder, self-harm/suicide ideation, substance use disorders (SUD), and sleep disorders. The secondary outcome was antidepressant use. Patients with prior psychiatric diagnoses or antidepressant use before the study window were excluded. Outcomes were assessed at 6-month, 1-year, 2-year follow-up time points, with statistical significance set at *p*<.05.

**Results:**

After matching, each cohort included 5488 patients (electrical vs flame), 5475 (electrical vs scald), and 35 554 (scald vs flame). At 6 months, electrical burns carried higher risk than flame for anxiety, PTSD, sleep disorders, adjustment disorder, and antidepressant use, while depression and substance use disorder were lower; these trends persisted at 1 and 2 years, with later reductions in suicidal ideation/self-harm. Compared with scald burns, electrical injuries showed consistently higher anxiety, PTSD, sleep disorders, adjustment disorder, and antidepressant use across all timepoints, with lower substance use disorder and self-harm emerging at later follow-up. Scald burns demonstrated the lowest psychiatric risk, with significantly lower anxiety, depression, PTSD, substance use disorder, and antidepressant use compared with flame burns throughout follow-up.

**Conclusions:**

Burn etiology is associated with distinct patterns of psychiatric morbidity, with electric burns linked to the highest risk across multiple outcomes and flame burns showing higher risk than scald burns. These results indicate correlations rather than causation and warrant prospective studies to confirm these associations and explore underlying mechanisms.

**Applicability of Research to Practice:**

Identifying psychiatric risk profiles by burn etiology can guide clinicians toward etiology-specific screening and timely mental health interventions, helping optimize recovery and long-term quality of life for burn survivors.

**Funding for the study:**

This study was supported by foundation funding through the Institute for Translational Sciences (UL1 TR001439), funded by the National Center for Advancing Translational Sciences at the NIH.

